# The Immunomodulatory Potential of Copper and Silver Based Self-Assembled Metal Organic Biohybrids Nanomaterials in Cancer Theranostics

**DOI:** 10.3389/fchem.2020.629835

**Published:** 2021-01-27

**Authors:** Neela Prajapati, Anik Karan, Elnaz Khezerlou, Mark A. DeCoster

**Affiliations:** ^1^ Department of Biomedical Engineering, Louisiana Tech University, Ruston, LA, United States; ^2^ Institute for Micromanufacturing, Louisiana Tech University, Ruston, LA, United States

**Keywords:** Copper/silver nano particles, nitric oxide, glioma, brain microvascular endothelial cells, tumor microenvironment, inflammatory stimulus

## Abstract

Copper high aspect ratio structures (CuHARS) and silver cystine nanoparticles (AgCysNPs) are two unique micro/nano particles under study here that show extensive anti-cancer effects on a glioma tumor cell line. These micro/nano particles have shown potent toxicity in the presence of inflammatory stimulus (combination of tumor necrosis factor, [TNF] and lipo-polysaccharide, LPS). CuHARS with a concentration of 20 μg/ml uniquely increased the catalytic generation of nitric oxide (NO), an important contributor in the immune system. This NO was generated in a cell culture tumor microenvironment (TME) in the presence of 25 µM S-nitrosothiol (cysteine-NO) and the inflammatory stimulus. CuHARS increased the NO production by 68.75% when compared to untreated glioma cells with CysNO and inflammatory stimulus. The production of NO was significantly higher under similar circumstances in the case of normal primary structural cells like brain microvascular endothelial cells (BMVECs). The production of NO by BMVECs went up by 181.25% compared to glioma cells. This significant increase in the NO concentration could have added up to tumorigenesis but the anti-cancer effect of CuHARS was prominent enough to lower down the viability of glioma cells by approximately 20% and increased the metabolism of structural cells, BMVECs by approximately 200%. The immunomodulatory effect of NO in the TME under these circumstances in the presence of the novel micro/nano material, CuHARS has risen up compared to the effect of inflammatory stimulus alone. The potency and specific nature of these materials toward tumor cells may make them suitable candidates for cancer treatment. Successive treatment of CuHARS to glioma cells also proved to be an effective approach considering the decrease in the total count of cells by 11.84 fold in case of three successive treatments compared to a single dose which only decreased the cell count by 2.45 fold showing the dose-dependent increasing toxicity toward glioma cells. AgCysNPs are another potent nanomaterial which also proved its significant toxic nature toward tumor cell lines as demonstrated here, but their immunomodulatory response is still unclear and needs to be explored further.

## Introduction

Functionalized metallic nanoparticles based on iron, copper, silver and gold have been in the field of cancer diagnostics and therapy for decades and have been providing alternatives to some conventional cancer treatment protocols. Silver nanoparticles have been observed to have excellent cytotoxic effect on MCF-7 breast cancer cell lines as observed by Kumar et al. (2018). Copper based nanomaterials, such as functionalized copper oxide and copper sulfide nanoparticles, have been under study for cancer therapy for prostrate and breast cancer studies in the last decade and have shown promising theranostic features (Liu et al., 2019; Zhou et al., 2016), but these were not studied in relation to their immunomodulatory potential in cancer theranostics. The concerned nano/micro-materials used in this project are copper high aspect ratio structures (CuHARS) and silver cystine nanoparticles (AgCysNPs) (Karekar et al., 2019). The two major constituents in CuHARS are copper and the amino acid dimer cystine (Deodhar et al., 2014) whereas for AgCysNPs, they are silver and cystine. Once synthesized, the CuHARS have the following benefits: 1) they scale from the nanometer to micrometer dimension in length, 2) the copper component of the biocomposite assists in imaging via microscopy (Cotton Kelly et al., 2015), and 3) the negative surface charge of the material with its high aspect ratio helps in functionalization with various assembly techniques. CuHARS are reported to have −28 to −33 mV zeta potential or surface charge and in case of AgCysNPs, zeta potential is around −16 to −23 mV (Karekar et al., 2019). Compared to CuHARS, the AgCysNPs are completely different in their structure and dimensions with oval shape and a size distribution of 20–80 nm (Karekar et al., 2019), but they also have negative surface potential like CuHARS mostly because the cystine here acts as a capping agent sitting on top of the silver. It has already been reported in many research studies about the toxic responses of silver and copper-based nano/micromaterials in biological environments (Ferdous and Nemmar, 2020; Karlsson et al., 2008). In the case of cancer studies, one of the objectives during synthesis of these kind of nano/micromaterials is to make them less toxic toward structural cells and to make them selectively toxic toward cancer cells.

Cancer is an uncontrolled growth of abnormal cells, which is also referred to as malignancy. Cancer has been tagged as the second leading cause of mortality in the United States and the current demographic says one out of six deaths worldwide has been tagged as an outcome of cancer (Seigel et al., 2014). Most of the conventional treatments for cancers include radiation therapy, chemotherapy, immunotherapy, and surgical exclusion of cancerous growths. In the modern era of therapeutics and pharmacology, targeted delivery of treatment drugs has been one of the hot topics for treatment of cancer due to the advancements of nanotechnology (Kumar, 2010; Mamelak and Jacoby, 2007; Mehta et al., 2008), but many challenges remain (Rosenblum et al., 2018).

According to the National Institutes of Health, the number of new brain and other nervous system related cancer cases in the year 2019 is estimated to be approximately 24,000, which is a 1.4% increment in the total number of recorded cases and the death toll related to brain cancer in 2019, which was approximately 18,000 (NIH National Cancer Institute, 2018). The primary brain tumors originate in the brain, and they are often not caused because of metastasis from other parts of the body. The most common primary brain tumors are gliomas, meningiomas, vestibular schwannomas, pituitary adenomas, and primitive neuroectodermal tumors (medulloblastomas) (WebMD, 2020). The term glioma includes glioblastomas, oligodendrogliomas, astrocytomas, and ependymomas. In this project, gliomas were studied and the effect of different nano/micromaterials on them was the primary area of focus. It has been reported by Karekar et al. in their study about the toxic responses of AgCysNPs and CuHARS specifically on the glioma cells used in the current study and the neuroblastomas (Karekar et al., 2019). The current study focuses on the potential of CuHARS in catalyzing NO, a key contributor of immune response in TME (Arora et al., 2018; Wink et al., 1998; Wink et al., 2011) and during normal physiological condition emulated by use of S-nitroso thiols, normally found in blood and during inflammation so as to study the effect in inducible NO (iNO) which is known to provide flexibility in dealing with immune challenges (Wink et al., 2011). The TME comprises both the tumor cell and the nearby blood supplying vessels with structural cells like endothelial cells which are active cells in the NO synthesis mechanism. The toxic effect of the nano/micromaterials on cancer cells has been contrasted here with their biofriendly nature toward surrounding structural cells, the brain microvascular vascular endothelial cells or BMVECs (Navone et al., 2013) which are the major cells in the blood brain barrier (BBB) (Daneman and Prat, 2015) as was observed by Karekar et al. (2019) and Darder et al. (2020) in their studies.

We have previously shown that CuHARS can work as catalysts in the synthesis of NO from S-nitrosothiols due to the presence of copper (Darder et al., 2020). NO is normally synthesized by oxidation of l-arginine by nitric oxide synthase (NOS), including inducible (iNOS) forms of the enzyme (Clancy et al., 1998). We also know that introduction of many foreign materials in a biological system can injure the cells which in turn excites inflammatory response resulting in formation of NO which works as a vasodilator promoting more influx of blood in that region and this may affect changes including susceptibility for autoimmunity (Gold et al., 1997). However, in some cases, it has been shown that NO can have an immunosuppression effect in the tumor microenvironment (TME) (PeÑarando et al., 2019). The influx of blood supply actually is supported by increasing concentration of white blood cells (WBC) in the specific region promoting the immune related responses. On the other hand, NO is also an angiogenic factor which also participates in tumorigenesis as observed in some studies (Gallo et al., 1998). NO is also known to activate very specific signal transduction pathways in endothelial cells, tumor cells and monocytes which is dependent on its concentration level (Wink et al., 2011). So, this complex system needs to be explored when it comes to the introduction of new micro/nano materials in cancer theranostics. The simulated effect of inflammatory responses through addition of TNF and LPS has been previously used (Jacobs, 1981; Clark et al., 2005; Gollahon et al., 1983; Yan et al., 2018) and was used here as well *in vitro*. A better understanding of the immunomodulatory responses can be obtained by including NOS inhibitors like l-NAME (Lo et al., 2016) in the similar TME under study which will serve as a control and show the resulting responses in the tumor growth regulation through the suppression in NO synthesis.

Another widely known aspect of tumor or cancer treatment is the dosage of the cancer theranostic drug and in most of the cases, under conventional treatment protocols, multiple dosage of drug administering is required for better efficacy (Gray et al., 2019; Peper, 2009). So, the effect of successive treatments is another area of interest whenever there is a new cancer theranostic protocol under study. In most of the cases of chemotherapy, a dose dependent, successive treatment has proven to be more effective provided there are widely known side-effects of chemotherapy. It was therefore the objective of this study to test the potential of CuHARS and AgCysNPs to decrease growth of cancer cells in direct comparison to normal cells, and to test these effects under normal cell culture conditions as well as in the inflammatory state. Considering the positive results achieved in the work presented here *in vitro*, this sets up the potential for future combination therapies using micro/nano materials like CuHARS and AgCysNPs in the field of cancer theranostics with reduced side effects on normal cells.

## Materials and Methods

**SCHEME 1:**
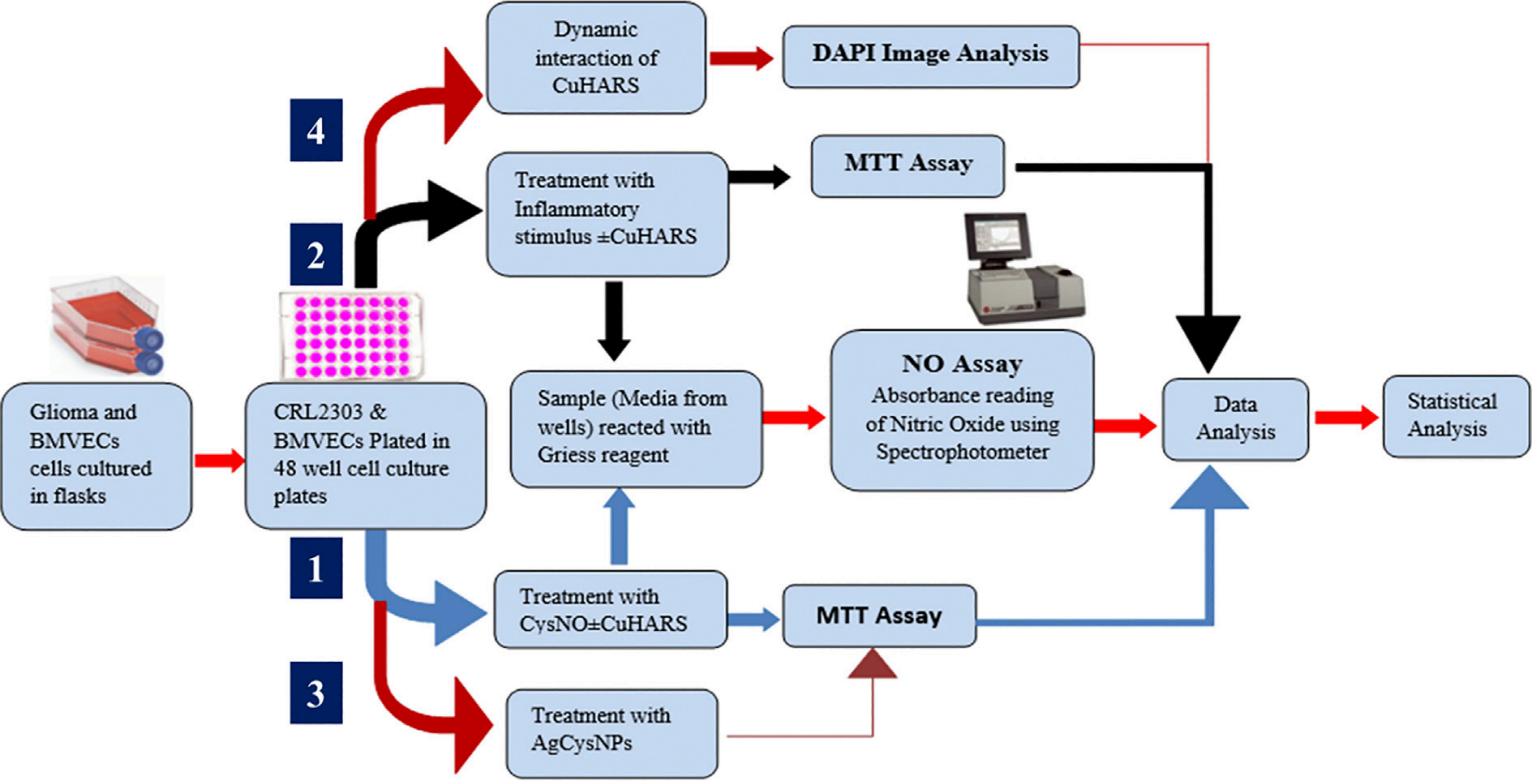
Schematics showing the overall experimental layout of the project illustrating the pathways for cell treatment with CuHARS in presence of CysNO (Darder et al., 2020) and in the presence of inflammatory stimulus, dynamic interaction of CuHARS with cells, and for treatment of cells using AgCysNPs.

An overview of the general methodology and workflow of the project is shown in Scheme 1.

### CuHARS and AgCysNPs Synthesis

CuHARS were synthesized from copper(II) sulfate and l-cystine at 37°C through the process of self-assembly following the protocol provided by Karekar et al., where l-cystine was dissolved in 0.1 M NaOH having a concentration of 72.9 mg/ml and 2 mg/ml aqueous solution of copper(II) sulfate was added to it (Karan et al., 2018; Karekar et al., 2019). Synthesized CuHARS were concentrated and dried for experimental usage after first destroying precipitates of copper not associated with CuHARS with brief 0.1 M HCl treatment and washing with sterile water as described (Karekar et al., 2019).

**FIGURE 1:**
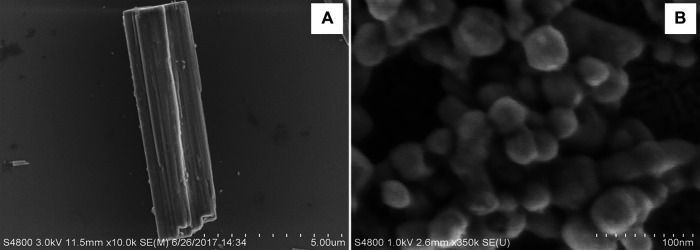
Scanning electron microscope images showing the significant differences in shape and size of CuHARS (Panel A) and AgCysNPs (Panel B). Scale bar in **(A)** = total length of 5.00 microns with 500 nm gradations indicated and scale bar in **(B)** = 100 nm length with 10 nm gradations.

AgCysNPs were synthesized using the similar protocol of self-assembly following the protocol provided by Karekar et al. where silver sulfate aqueous solution was prepared having a concentration of 2 mg/ml. l-cystine dissolved in 0.1 M NaOH (conc. 72.9 mg/ml) was warmed at 37°C and silver sulfate solution was added to it and stored at 37°C for 24 h (Karekar et al., 2019). The AgCysNPs formed were concentrated, dried and weighed for experimental purpose. Using scanning electron microscopy (SEM), the stark differences in morphology and size of the synthesized CuHARS and AgCysNPs were compared as shown in Figures 1A,B, respectively.

### Cell Culture

**FIGURE 2:**
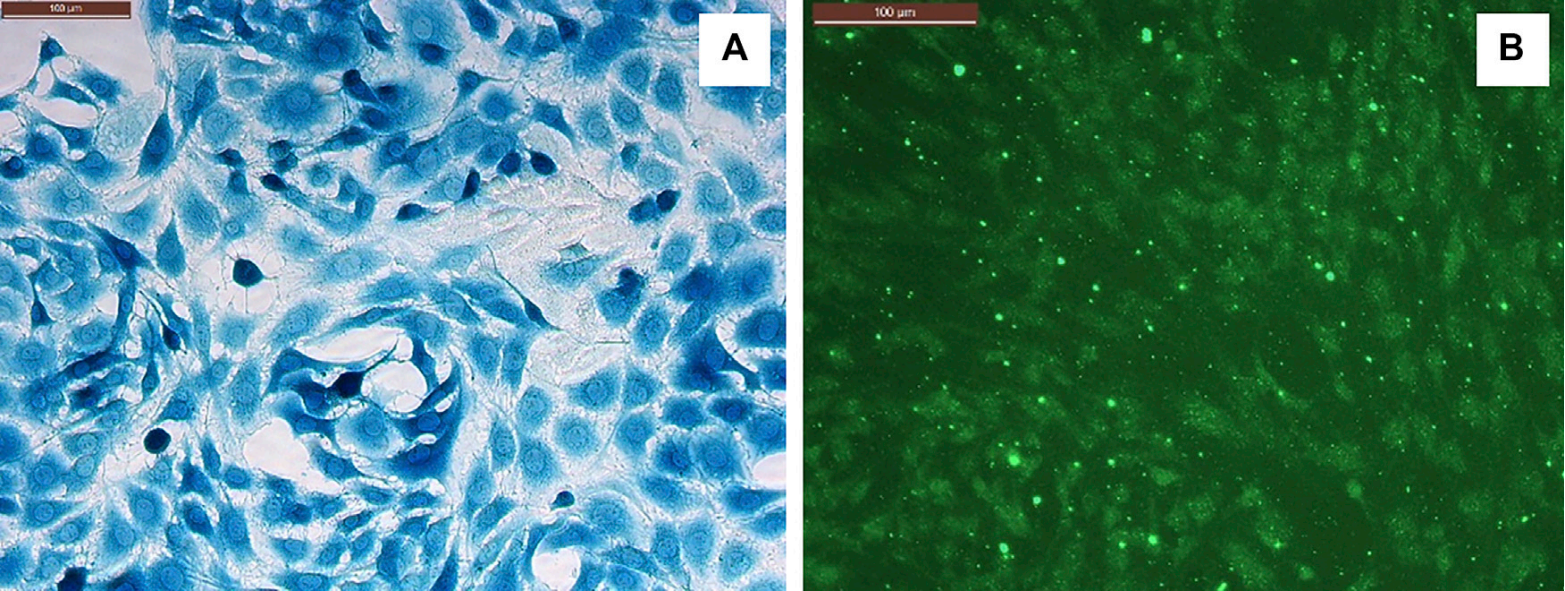
**(A)** Glioma cells (CRL2303) stained against β-galactosidase gene. **(B)** BMVECs stained against VWF. Scale bar (upper left in both panels) = 100 microns.

CRL2303(ATCC) glioma cells were cultured in a complete growth medium containing DMEM (ATCC), 10% Fetal Bovine Serum (Sigma-Aldrich), penicillin/streptomycin, and 1% MEM Non-Essential Amino Acid solution (Sigma) (Joshi et al., 2012). Cells between passages P8 to P20 were used for experiments. BMVECs were isolated from cortical tissue obtained after cervical disarticulation of Sprague-Dawley rat pups (2–3 days). All procedures were performed adhering to the protocol approved by the Louisiana tech University Animal Care and Use Committee. The cortical tissue was processed as described previously by Wang et al. (2005) to obtain a primary glial culture which was purified by treating with 5.51 µM of puromycin dihydrochloride (Millipore Sigma, St. Louis, MO, USA) and cultured in Rat Endothelial Growth Medium (Cell Applications Inc.) with 6% of Rat Endothelial Growth Factor (Cell Applications Inc.) to obtain a pure BMVECs culture (Abbott et al., 1992; Saleh et al., 2020). Both glioma (CRL2303) and endothelial (BMVECs) cells were cultured *in vitro* at 5% CO_2_ and 37°C. The glioma cells were characterized using β-gal staining (Jäkel and Dimou, 2017) using the β-galactosidase Reporter Gene Staining Kit (Sigma-Aldrich) and BMVECs were characterized by staining them against Von Willebrand factor, a blood clotting protein specific to endothelial cells, using VWF related antigen (Santa Cruz biotechnology, CA) (Wang et al., 2012) as shown in Figure 2.

### CuHARS Treatment in Combination With CysNO

Glioma and endothelial cells were plated in 48 well cell culture plates at the density of 10,000 per ml. When the confluency of cells reached 60%, the cells were treated with 0.1 mM of S-nitrosocysteine (CysNO), a nitric oxide precursor found in blood, which was freshly prepared before each experiment as described by Harding and Reynolds (Harding and Reynolds, 2012). Cystine (0.1 mM) was mixed with 0.1 mmol of tert-butyl nitrite in 2 ml of water set up in an ice bath in a stirrer for 30 min to generate CysNO. Three different concentrations of CysNO, namely, 25, 50, and 100 µM, for glioma cells, and 10, 25 and 50 µM for BMVECs, were chosen to obtain NO release profile using 10 or 20 μg/ml of CuHARS. CuHARS were added immediately after CysNO to the cells which were then incubated at 37°C in 5% CO_2_ for 3 h.

### CuHARS Treatment in Combination With Inflammatory Stimulus

The cells plated at 10,000 per ml in 48 well cell culture plates were treated with an inflammatory stimulus, a combination of 100 ng/ml of TNF and 5 μg/ml of LPS in the presence and absence of 20 μg/ml of CuHARS when the cells reached 30% confluency. The glioma cells were incubated at 37°C and 5% CO_2_ for 48 h after treatment to assess the NO release in the cells due to the inflammatory stimulus, while BMVECs were incubated up to 96 h. The NO assay was performed twice on BMVECs at 48 and 96 h after treatment to compare NO release at the same time point (48 h) and at approximate confluency (96 h) as was performed for glioma cells.

### Nitric Oxide Assay

Nitric oxide collected in the wells after incubation with their respective treatment conditions were reacted with Griess Reagent for nitrite (a stable breakdown product of NO) determination as described in the protocol (Invitrogen, G-7921) (Ashpole et al., 2014). The absorbance values of the solution obtained were read using a spectrophotometer (Beckman Coulter DU 800) at 548 nm. For each experiment, a standard curve with four to five standard solutions of nitrite (0, 5, 25, 50, and 75 µM) obtained from the reagent kit were prepared which was used to convert the absorbance values into NO concentrations.

### Silver Nanoparticle (AgCysNPs) Treatment on Cells

Both glioma (CRL2303) and endothelial cells (BMVECs) were plated at 10,000 per ml cell density and treated with 100, 250 and 500 ng/ml of AgCysNPs when they reached 60% confluency. BMVECs were treated with an additional concentration of 2 μg/ml to determine the toxic concentration of AgCysNPs to normal endothelial cells. The cells were incubated for 24 h with AgCysNPs at 37°C and 5% CO_2_ before toxicity due to the treatment was assessed using MTT assay.

### Cell Interaction Studies With Multiple Treatment Dosages of CuHARS

The glioma cells were plated into a 48 well plate at the density of 3,000 cells per ml and incubated at 37°C and 5% CO_2_. To test the effect of dynamics interaction of CuHARS over time, glioma cells were exposed to one, two and three subsequent treatments of 10 μg/ml of CuHARS, each at the interval of 24 h. The effect on the cells was analyzed by fixing the cells using Diff-Quik fixative (methanol), after their respective period of incubation with CuHARS. The fixed cells were stained with 4,6-diamidino-2-phenylindole (DAPI) to analyze for cell number and cell viability. Three images for each well were taken using a Leica DMI 6000B inverted microscope for the cells stained with DAPI and analyzed for the number of cells and total area coverage by nuclei of cells per image using Image Pro-Plus version 7.0 (Media Cybernetics, Rockville, MD). Comparison of cytotoxicity of CuHARS in glioma cells with a well-known anti-cancer drug doxorubicin(DOX) was carried out to show the potential relevance of the nanomaterials usage in the field of cancer theranostics.

### MTT Assay

The MTT (3-(4,5-dimethylthiazol-2-yl)-2,5-diphenyl tetrazolium bromide) assay that estimates the cell viability by comparing cellular metabolic activity, was performed as reported previously (Rodrigues et al., 2020; Vivès et al., 1997) as a measure to determine cytotoxicity. Cells were incubated with MTT solution (1.25 mg/ml of MTT powder in RPMI medium) for 1 h at 37°C. The formazan crystals formed after incubation were dissolved in 91% isopropyl alcohol and absorbance reading was obtained using a Thermoscan Plate Reader at wavelength of 570 nm. For CuHARS and CysNO treatment to cells, MTT assay was performed after 24 h of treatment while for the cells treated with CuHARS and inflammatory stimulus, it was performed soon after NO assessment at 48 h for glioma cells and 96 h for BMVECs.

### Statistical Analysis

Statistical analysis was performed using one-way ANOVA with the multiple comparison method for experiments consisting of more than two groups and two-tailed t-tests for comparison between two groups. The results were considered statistically significant when *p* < 0.05. Data are presented as the mean ± standard error of mean (SEM) of *n* = 9 wells for each experiment.

## Results

### Treatment of Glioma Cells With CuHARS in the Presence of CysNO

**FIGURE 3:**
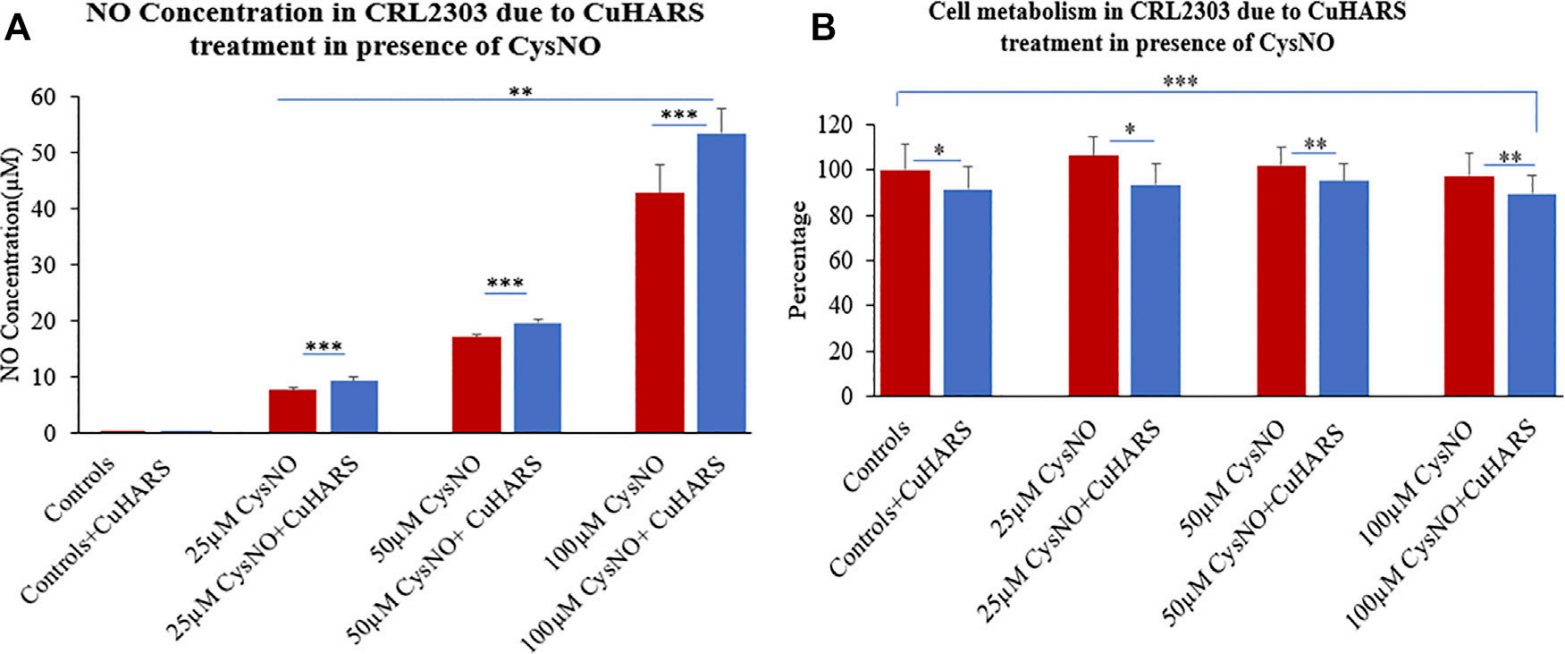
CRL2303 glioma cell response to 20 μg/ml CuHARS treatment in presence of three different concentrations (25, 50 and 100 µM) of CysNO, an NO precursor normally found in blood. **(A)** NO concentration in the cells due to the treatment showing significant increase in NO release from CRL2303 cells treated with CuHARS compared to cells treated with CysNO alone. **(B)** Cell metabolism due to the treatment showing inhibition of cell metabolism for CuHARS treated cells. Data represent average of three experiments (*N* = 3) with triplicated wells (*n* = 3). The error bars represent SEM values, “***” represents *p* < 0.001, “**” represents *p* < 0.01 and “*” represents *p* < 0.05.

Concentration dependent increase in nitric oxide synthesis was observed in the wells with glioma cells treated with CuHARS in the presence of CysNO to a greater extent than for the cells treated with the same amount of CysNO alone (Figure 3A). A treatment of 20 μg/ml of CuHARS for 3 h in the presence of 25 and 50 µM CysNO increased the mean NO concentration in the wells by 1.6 ± 0.6 and 2.6 ± 0.6 µM each compared to the cells treated with same amount of CysNO alone (Figure 3A). This increase in NO went significantly higher with a mean increase of 10.7 ± 4.5 µM when treated with the same amount of CuHARS in the presence of 100 µM CysNO. The glioma control cells that were treated with media alone and with CuHARS alone did not produce any significant NO in the wells.

### Cytotoxicity due to CuHARS and CysNO Treatment on Glioma Cells

After determination of NO concentration due to 20 μg/ml of CuHARS in the presence of CysNO, cellular metabolism for all the treatment conditions was quantified using MTT assay at 24 h after treatment. CuHARS have shown concetration dependent cytotoxicity in earlier studies in glioma cells (Karekar et al., 2019). During this treatment we observed 10% decrease in cell metabolism for the control cells treated with CuHARS alone (20 μg/ml) compared to non-treated cells. The cells treated with 25, 50 and 100 µM CysNO alone showed a slight increase in cell metabolism at lower concentration (25 µM) which went on decreasing slightly with higher concentrations used. This increase in cell metabolism by CysNO was reversed by the presence of 20 μg/ml CuHARS which inhibited the metabolism by 13, 7 and 8% for cells treated with 25, 50 and 100 µM CysNO respectively (Figure 3B). As CuHARS is a slowly degrading material the viability for glioma cells goes on decreasing with increasing time points due to toxicity imparted by the release of copper from CuHARS as shown in Karekar et al. (2019), and further proved by following sections in this paper.

### Treatment of TNF/LPS Stimulated Glioma Cells With CuHARS

**FIGURE 4:**
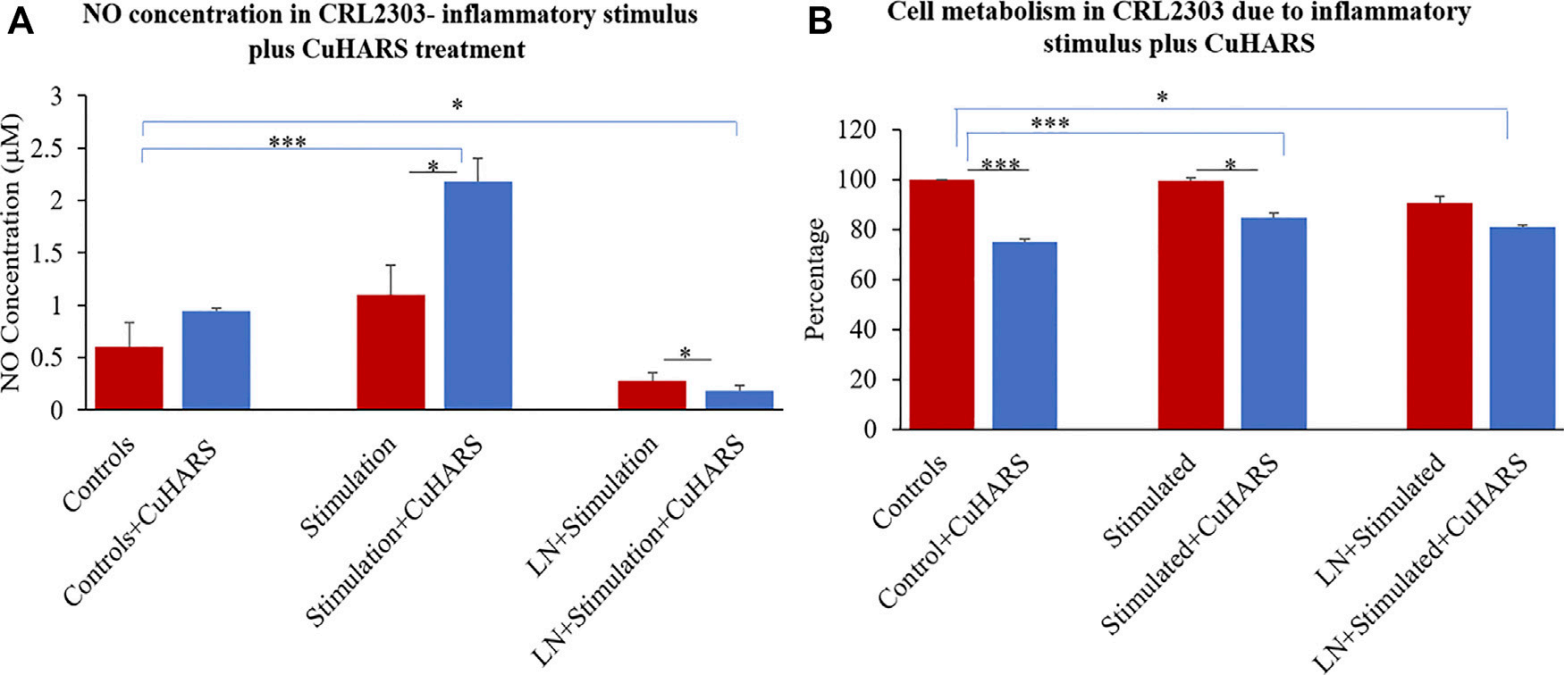
CRL2303 glioma cells response to 20 μg/ml CuHARS treatment in presence of inflammatory stimulations (100 ng/ml of TNF+ 5 μg/ml of LPS). **(A)** NO concentration in the cells due to the treatment showing significant difference in NO release from CRL2303 cells treated with CuHARS compared to cells treated with inflammatory stimulus alone. The negative controls treated with LNAME (LN), a NOS inhibitor, showed significant inhibition in NO release. **(B)** Cell metabolism due to the treatment showing inhibition of cell metabolism for CuHARS treated glioma cells including in the presence of inflammatory stimuli (as in Panel A). Data represent average of three experiments (*N* = 3) with triplicated wells (*n* = 3). The error bars represent SEM values, “***” represents *p* < 0.001, and “*” represents *p* < 0.05.

A two-fold increase in NO synthesis (2.2 µM) was observed in glioma cells treated with inflammatory stimulus, a combination of 100 ng/ml of TNF and 5 μg/ml of LPS, in the presence of 20 μg/ml of CuHARS compared to the cells treated with the inflammatory stimulus alone (1.1 µM), 48 h after the treatment (Figure 4A). Treatment with inflammatory stimulus resulted in a modest increase of 0.5 ± 0.2 µM of NO while the combinatory treatment of the stimulus and CuHARS led to an increase of 1.6 ± 0.2 µM compared to the non-treated control cells. The addition of (N(ω)-nitro-l-arginine methyl ester (l-NAME), an NOS inhibitor to the system, resulted in significant inhibition of the NO produced in the wells, thus validating this observation of NO increase due to CuHARS as measured via the enzyme NOS.

### Cytotoxicity due to CuHARS and Inflammatory Agent Treatment on Glioma Cells

**FIGURE 5:**
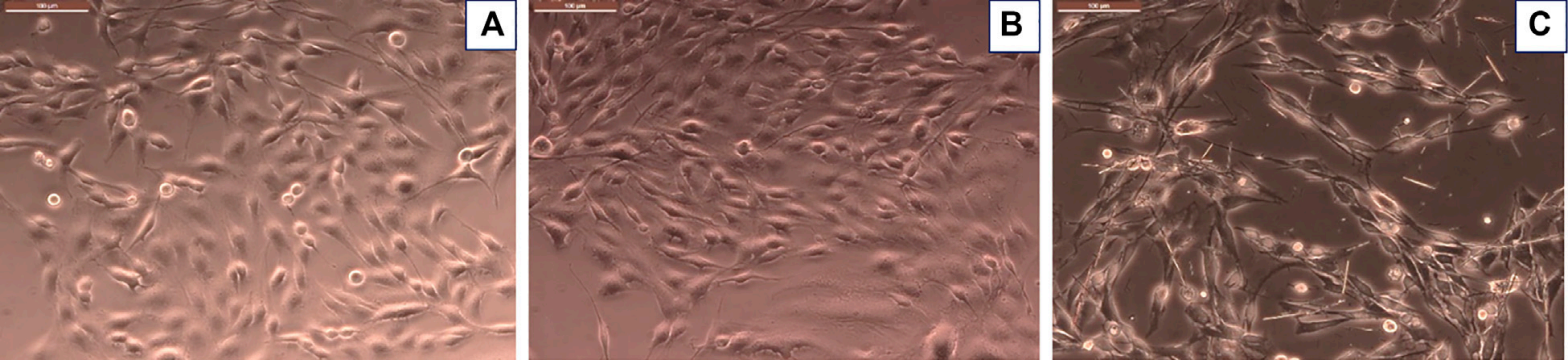
Glioma cells treated with inflammatory stimulus in presence of 20 μg/ml of CuHARS causes significant toxicity to the cells. **(A)** Control cells treated with media alone. **(B)** Cells treated with inflammatory agents. **(C)** Cells treated with inflammatory agents and 20 μg/ml of CuHARS, Scale bar = 100 µm (upper left of each figure); Magnification = ×200.

Morphological evaluation of cells treated with inflammatory stimulus or with inflammatory stimulus plus CuHARS supported metabolic biochemical assay results (Figure 5). Using inverted phase microscopy, CuHARS could be observed in cultures treated with 20 μg/ml of the materials (Figure 5C), and when combined with inflammatory stimulus, this diminished cell number compared to control wells (Figure 5A).

An MTT assay was performed after 48 h of treatment to quantify cellular metabolism of glioma cells with no treatment (controls) and with treatment of inflammatory stimulus in presence or absence of 20 μg/ml of CuHARS to determine the cytotoxicity due to the treatment. The assay indicated no significant difference in cell metabolism for glioma cells treated with inflammatory agents computed by comparison to the control conditions (Figure 4B). In contrast, metabolism of cells treated with CuHARS went down in all conditions treated with CuHARS including in the presence of inflammatory stimuli (Figure 4B). The cytotoxicity due to the treatment was explicitly visible under the microscope as shown by images captured using Leica DMI 6000B microscope (Figure 5). For the cells treated with inflammatory agents in the presence of CuHARS, metabolism went down by 15% only which can be compared to increased metabolic activity due to treatment with inflammatory agents in other cell types (Jacobs, 1981; Yan et al., 2018). Suppression of NO release on cells using l-NAME, led to decreased cell metabolism by 10% which was further suppressed by another 10% (overall 20%) due to the use of CuHARS.

### Treatment of Primary BMVECs With CuHARS in Presence of CysNO

**FIGURE 6:**
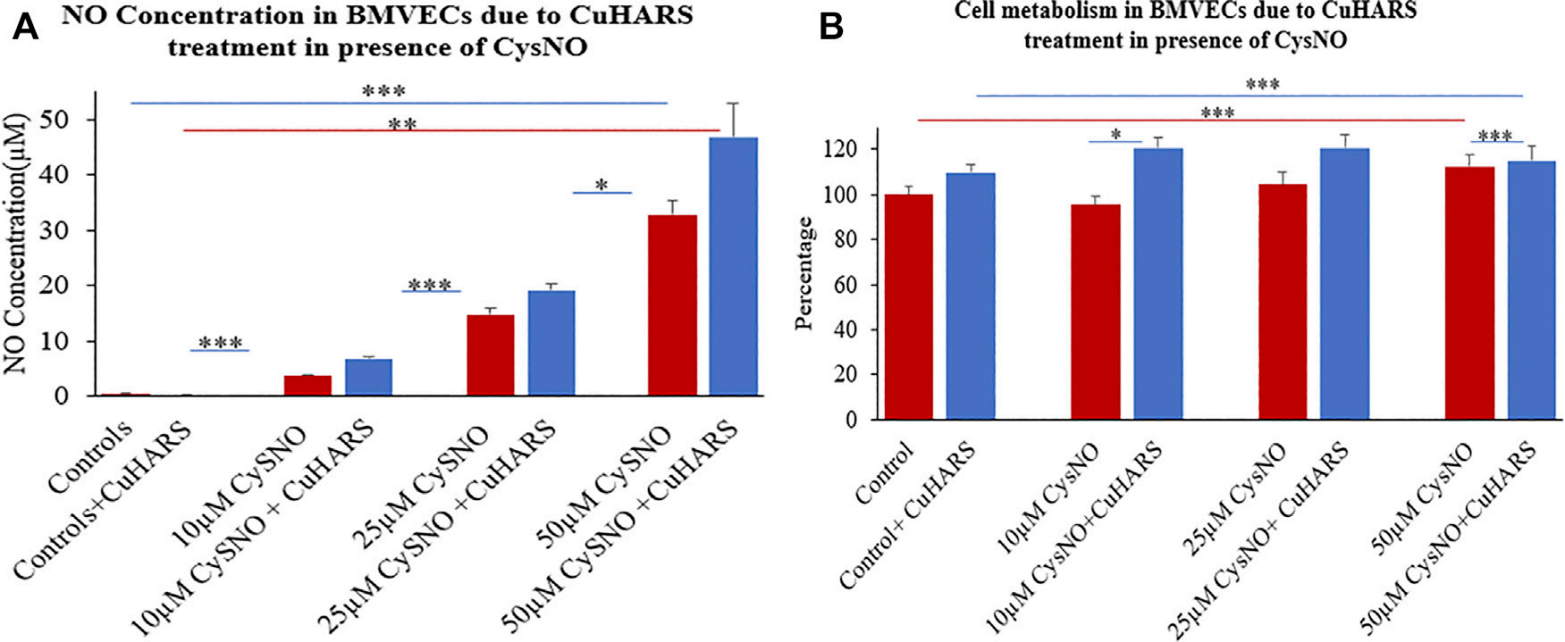
Normal brain endothelial cell (BMVECs) response to 20 μg/ml CuHARS treatment in presence of three different concentrations (10, 25 and 50 µM) of CysNO. **(A)** NO concentration in the BMVECs due to the treatment showing significant increase in NO release from cells treated with CuHARS compared to cells treated with CysNO alone. **(B)** Cell metabolism due to the treatment showing increase in cellular metabolism for CuHARS treated cells compared to when treated with CysNO alone. Data represent average of three experiments (*N* = 3) with triplicated wells (*n* = 3). The error bars represent SEM values, “***” represents *p* < 0.001, “**” represents *p* < 0.01 and “*” represents *p* < 0.05.

Similar to glioma cells, primary BMVECs treated and non-treated with CuHARS alone showed no significant NO synthesis in the wells (Figure 6). However, a treatment of 10 and 20 μg/ml of CuHARS in presence of 10, 25 and 50 µM of CysNO yielded a concentration dependent increase in NO release from the BMVECs. For 10 and 25 µM of CysNO treatment, 20 μg/ml of CuHARS treatment yielded an increase of 3 ± 0.2 and 4.5 ± 1.1 µM NO concentration in the wells compared to CysNO alone, while for 50 µM of CysNO the NO concentration went significantly higher due to CuHARS to an additional 14 ± 3.5 µM of NO compared to CysNO alone (Figure 6A). A similar trend was observed with lower concentration (10 μg/ml) of CuHARS for the same concentrations of CysNO tested, giving lower value of NO increase compared to 20 μg/ml of CuHARS (Supplementary Figure S1). This result shows that the increase in NO concentration in the wells depends on both the concentration of CuHARS and CysNO used but the reaction is limited only due to the availability of CysNO (Darder et al., 2020) as it is a transient molecule which degrades quickly (within few hours) while CuHARS degrades slowly and can remain active over considerably very long period of time (few days) (Deodhar et al., 2014; Karekar et al., 2019).

An interesting observation in the experiment is that the NO yield at 50 µM CysNO for BMVECs is higher than that observed for glioma cells (Figure 3A), hence indicating that glioma cells can produce NO at reduced level compared to the endothelial cells which are capable of constitutive NO synthesis due to the presence of endothelial nitric oxide synthase (eNOS) that can contribute to the major part of NO production in the vascular system (Zhao et al., 2015).

### Lack of Cytotoxicity due to CuHARS and CysNO Treatment on BMVECs

In contrast to the glioma cells, the MTT assay of CysNO treatment on BMVECs in presence of CuHARS showed no toxicity to the cells but indicated higher cell metabolism between 10 and 20% by 24 h of treatment. The cell metabolism increased by 10% for the control cells and by 25, 15 and 3% respectively in the presence of 10 μM, 25 and 50 µM CysNO when treated with 20 μg/ml of CuHARS compared to the cells treated with CysNO alone (Figure 6B). Copper is known to be angiogenic, and lower concentrations of copper have been shown to increase the viability and stimulate proliferation in BMVECs (Wang et al., 2016), human umbilical vein endothelial cells (HUVECs), (Hu, 1998; Li et al., 2012), and human pulmonary arterial endothelial cells (HPAECs), (Demura et al., 1998), but not in human dermal fibroblasts and smooth muscle cells (Hu, 1998). Copper stimulation of HUVEC and HPAEC cell metabolism is eNOS dependent (Demura et al., 1998; Li et al., 2012). BMVECs have characteristic eNOS expression unlike glioma cells and show enhanced NO production in the system due to CuHARS stimulation which we controlled here (Figures 6A, 3A). This set of treatments potentially results in higher BMVEC metabolism in contrast to the glioma cells.

### Treatment of TNF/LPS Stimulated BMVECs With CuHARS

**FIGURE 7:**
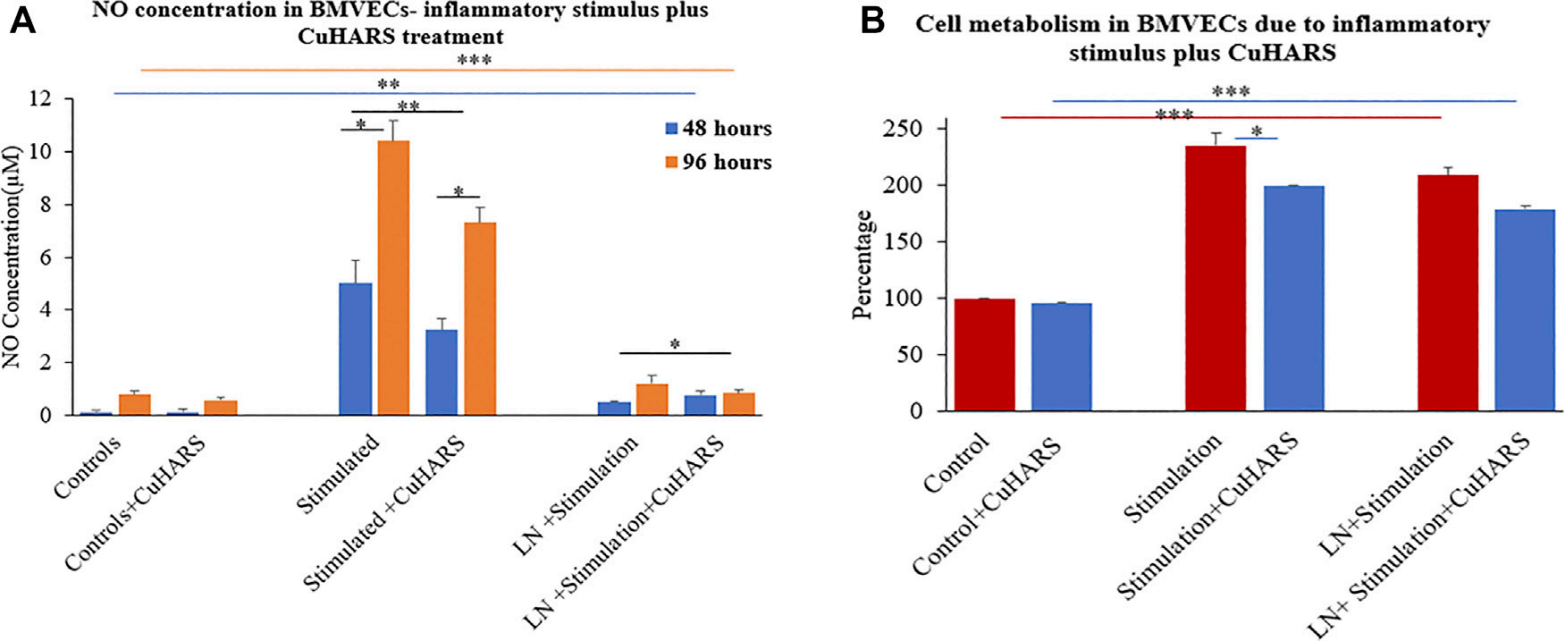
Normal brain endothelial cell (BMVECs) response to 20 μg/ml CuHARS treatment in the presence of inflammatory stimulations (100 ng/ml of TNF + 5 μg/ml of LPS). **(A)** NO concentration in the cells due to the treatment showing inhibition in NO release in both 48- and 96-h time point in BMVECs treated with CuHARS compared to cells treated with inflammatory stimulus alone. The negative controls treated with LNAME, an NOS inhibitor, showed significant inhibition in NO release. **(B)** Cell metabolism due to the treatment showing significant increase in cell metabolism in BMVECs treated with inflammatory stimulus compared to the controls and slight inhibition due to CuHARS in the cells treated with the combination of CuHARS and inflammatory stimulus. Data represent average of three experiments (*N* = 3) with triplicated wells (*n* = 3). The error bars represent SEM values, “***” represents *p* < 0.001, “**” represent *p* < 0.01 and “*” represents *p* < 0.05.

NO concentration was measured on the BMVECs with the same inflammatory stimuli (100 ng/ml TNF + 5 μg/ml LPS) as was used for the glioma cells. In line with the results observed for the glioma cells, the concentration of NO increased significantly for BMVECs supplied with inflammatory agents compared to the controls (Figure 7), but strikingly, the presence of CuHARS inhibited the NO release from these stimulated cells in contrast to the results observed for glioma cells in which CuHARS contributed to the increased NO synthesis. NO absorbance readings were taken at 48 and 96 h to resemble the exact time point (48 h) and approximate confluency (96 h) to the glioma cells, as BMVECs grow at moderate speed compared to the glioma cells. At 48 h, an increase in 4.9 ± 0.5 µM of NO was observed in BMVECs stimulated by inflammatory agents alone compared to the control cells which were inhibited by 1.8 ± 0.6 µM due to the presence of 20 μg/ml CuHARS and by 96 h these values went up to 9.6 ± 0.5 µM increase and 3.1 ± 0.7 µM inhibition respectively (Figure 7A). Treatment of wells with l-NAME resulted in significant inhibition of NO release in all conditions thus validating the measured release and inhibition profiles of NO in the cells as mediated by NOS enzyme.

### Cytotoxicity Due to CuHARS and Inflammatory Agent Treatment on BMVECs

MTT results for BMVECs treated with inflammatory stimulus showed more than two fold increase in cell metabolism compared to the controls at 96 h (Figure 7B). Metabolism for the cells treated with the combination of inflammatory stimulus and CuHARS showed slight inhibition compared to inflammatory stimulus alone, but very significant increase in metabolism compared to the controls with no treatment and treated with CuHARS alone.

### Silver Nanoparticles (AgCysNPs) Treatment on CRL2303 Glioma and BMVECs

**FIGURE 8:**
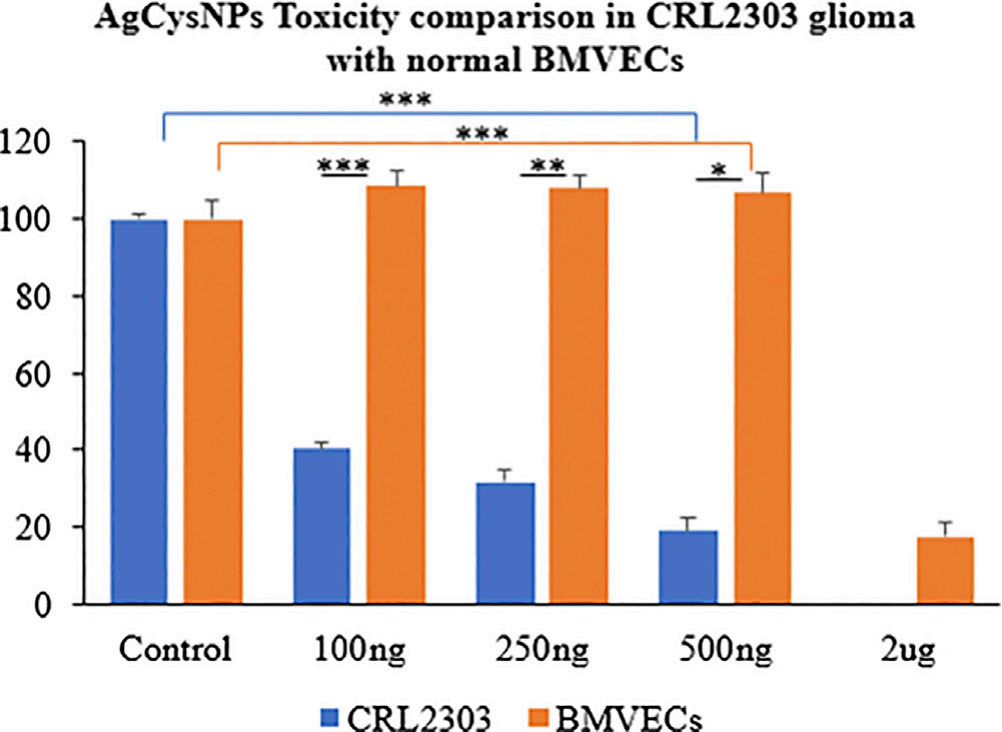
Toxicity comparison between CRL2303 glioma cells and normal BMVECs due to treatment with AgCysNPs showing high toxicity imparted by the silver-cystine biohybrid to glioma cells from concentration of 100–500 ng/ml but BMVECs requiring about four fold (2 μg/ml) more of the materials compared to glioma cells to show same level of toxicity as was observed for 500 ng/ml in glioma cells. Data represent average over three experiments (*N* = 3) with triplicated wells (*n* = 3). The error bars represent SEM values, “***” represent *p* < 0.001, “**” represent *p* < 0.01 and “*” represent *p* < 0.05.

MTT assay indicated significantly high cell viability for BMVECs incubated with AgCysNPs at concentrations of 100, 250, and 500 ng/ml per ml, in contrast to glioma cells which were killed by these concentrations (Figure 8). AgCysNPs showed concentration dependent toxicity for CRLS with 80% inhibition in cell viability at 500 ng/ml while it took four-fold (2 μg/ml) of the concentration of AgCysNPs to obtain the same level of inhibition in BMVECs (Figure 8).

### Dynamic Interaction Studies of CuHARS

**FIGURE 9:**
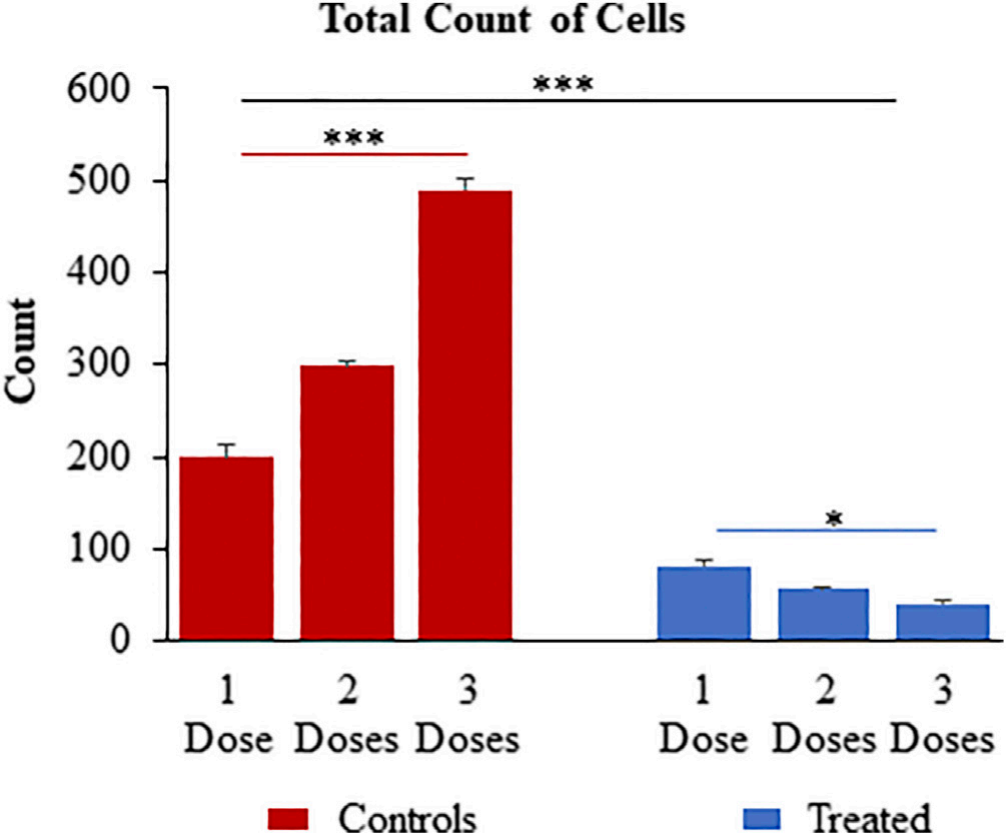
Successive treatment of CuHARS (10 μg/ml) on CRL-2303 glioma cells plated at 3,000 cells per ml at an interval of every 24 h up to three doses showing significant increase in toxicity on the glioma cells with increase in number of treatments as represented by the DAPI image analysis results for total count of cells. Red bars on left of panel are control wells and blue bars represent wells treated with CuHARS. Data represent average over three experiments (*N* = 3) with triplicated wells (*n* = 3). The error bars represent SEM values, “***” represent *p* < 0.001 and “*” represent *p* < 0.05.

**FIGURE 10:**
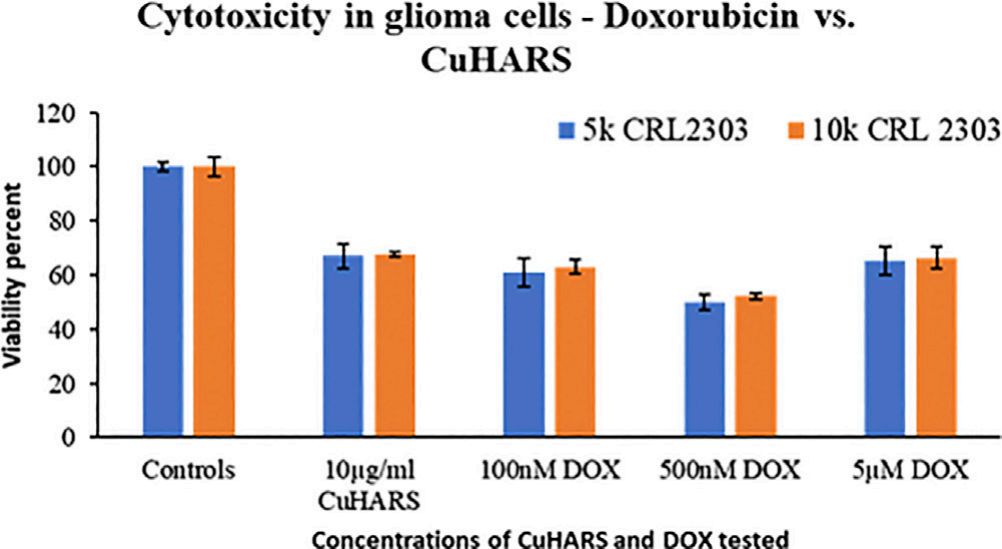
Comparison of the toxicity response of CuHARS and anti-cancer drug Doxorubicin (DOX), against glioma cells CRL2303, plated at 5,000 (5k) or 10,000 (10k) cells/well. Viability was calculated using MTT assay as described in methods, and data were normalized to control values, which were set to 100%. Data represent average over three experiments (*N* = 3) with triplicated wells (*n* = 3). The error bars represent SEM values.

The assessment of toxicity due to successive treatment of up to three doses of 10 μg/ml CuHARS each at an interval of 24 h, was quantified by staining for the cell nuclei using DAPI after termination of experiments at defined times (1 dose-24 h, two doses- 48 h and three does -72 h). DAPI images were taken using Leica DMI 6000B microscope (Supplementary Figure S2) and analyzed in Image Pro Plus image analysis software (version 7.0). The count of cells based on number of nuclei present per ROI was measured where an ROI represents an image randomly selected from the wells. Three ROIs per well were chosen for three experiments each with triplicated wells per condition. For untreated wells, the average number of cells increased over time, as the cancer cells continued to aggressively grow (Figure 9). CuHARs treatments suppressed all growth compared to control wells. The number of cells for one dose treatment decreased by 2.45-fold and two and three doses of treatment decreased it by 5.2 and 11.84-fold, respectively shown in Figure 9. As expected, the same trend was observed when we calculated total nuclear area based on these DAPI measurements (data not shown). Repeated progression of cancer is a very challenging aspect of cancer treatment (Cagan and Meyer, 2017; Califano and Alvarez, 2017). For example, when we directly compared CuHARS with the well-known anti-cancer drug doxorubicin (DOX), we found that 10 μg/ml of CuHARS worked similarly well to a range of DOX from 100nM to 5 µM (Figure 10). DOX seemed to show biphasic cytotoxicity response (Lopes et al., 2008; Koleini and Kardami, 2017) in glioma cells as 5 µM DOX showed lower inhibition in cell metabolism than 500 nM with cell metabolism approximate to 100 nM of DOX. Additionally, the values for inhibition of growth over the 50-fold range of DOX concentrations were all quite similar to each other (and to CuHARS), indicating the limits of efficacy of any one drug against aggressive cancer such as the glioma cells used here. The comparative results of DOX vs. CuHARS in cell killing were also verified by measurement of cell proliferation using DAPI image analysis (Supplementary Figure S3). These results demonstrate the potential of CuHARS in inhibition of progression of cancer using successive targeted treatments, and the limits of any one drug at this time. Using novel materials such as CuHARS may provide an important new strategy for impacting cancer inhibition and progression.

## Discussion

The catalysis of NO in the presence of CuHARS in a TME could be a significant factor in tumorigenesis, but in our study, it was clearly shown that the increase in NO concentration in TME can be easily negated by introduction of inflammatory stimulus along with CuHARS and thus, result in increased overall potency of killing toward glioma cell lines. This could become a new direction in immunomodulatory cancer theranostics, to balance the complex interplay between NO-induced increased blood flow which could provide growth factors to tumor cells, and the involvement of NO as an inflammatory stressor which could help kill the tumor cells. As shown here, the presence of the novel micro/nano materials CuHARS and AgCysNPs not only induces necrosis of tumor cells, but also increases the metabolic activity of surrounding structural cells like BMVECs which could give new insight into novel immunomodulatory pathways. The additive toxicity of CuHARS in the presence of inflammatory stimulus is strengthening the immunomodulatory pathways by building up more NO and introducing toxicity specifically toward cancer cells keeping the structural cells healthy and increasing their metabolic activity which is increasing the overall stability of the regulating biological system. In this regard it was interesting to note that while CuHARS potentiated the NO concentration in glioma cells treated with the donor CysNO (Figure 3A), under the same treatment conditions, the metabolism of those cancer cells was suppressed by CuHARS (Figure 3B). The control experiment using l-NAME in TME (glioma cells), clearly indicated that NO inhibition at the enzyme level can greatly diminish the effect of CuHARS in the system (Figure 4).

Another aspect of these micro/nano materials is their biodegradability and biocompatibility which gives them versatility in their applications related to other fields of biomedical engineering like wound healing (Darder et al., 2020). The excellent dose dependent response of CuHARS could potentially replace the usage of chemotherapeutic drugs or be used in combination with other treatments. This is important as shown under inflammatory conditions for normal BMVECs studied here where increasing concentrations of CysNO with CuHARS resulted in significant elevations in NO concentration (Figure 6A), as expected. However, these same combinations increased the metabolism of these normal BMVEC cells (for 10–25 µM CysNO), but within limits, as the combination of the highest stimuli (50 µM CysNO + 20 μg/ml of CuHARS) did not potentiate cell metabolism compared to 50 µM CysNO alone (Figure 6B). This demonstrates the well-known biphasic nature of many biological processes whereby a given stimulus by definition may increase outcomes with increasing concentrations of the stimulus, but within limits, as too much of a material cannot be fully processed by the organism, or in this case, cells (*in vitro*). In the case of cancer, development of novel, fully degradable nanomaterials are of interest as a carrier or direct delivery method for destroying diseased cells. As shown here, the comparative toxic effect of CuHARS and AgCysNPs indicates more toxic potency of AgCysNPs toward tumor cell lines (glioma) compared to CuHARS. We had previously shown that AgCysNPs were potently toxic toward cancer cells at the ng/ml level both when applied as a nanofilm layer interacting with cells as well as using traditional methods in solution as carried out here (Karekar et al., 2019). We have also provided evidence that these materials are less injurious to the normal, structural cells of the blood brain barrier, the BMVECs, compared to the target, glioma cells. While this result is encouraging for both the copper-containing CuHARS and silver-containing AgCysNPs, potential inflammatory influences when treating with our synthesized AgCysNPs remains to be explored.

## Data Availability Statement

The raw data supporting the conclusions of this article will be made available by the authors, without undue reservation.

## Ethics Statement

The animal study was reviewed and approved by Louisiana Tech University Institutional Animal Care and Use Committee (IACUC).

## Author Contributions

NP, AK, and MD designed, conducted, and analyzed the experiments; EK conducted and analyzed experiments on dynamic interaction studies of CuHARS; NP, AK, EK, and MD wrote the manuscript.

## Funding

This research was supported, in part, by NSF award #1547693 and the James E. Wyche III endowed professorship made available through the State of Louisiana Board of Regents Support Funds.

## Conflict of Interest

The authors declare that the research was conducted in the absence of any commercial or financial relationships that could be construed as a potential conflict of interest.
