# Supplementary material for: The Immunomodulatory Potential of Copper and Silver Based Self-Assembled Metal Organic Biohybrids Nanomaterials in Cancer Theranostics
Source: Front Chem. 2021 Jan 27;8:629835. doi: 10.3389/fchem.2020.629835 (PMC7873042; doi:10.3389/fchem.2020.629835)
Supplement: Supplementary file 1 [file datasheet1.pdf]

THE IMMUNOMODULATORY POTENTIAL OF COPPER AND SILVER BASED SELF-ASSEMBLED METAL ORGANIC BIOHYBRIDS (MOBS) NANOMATERIALS IN CANCER THERANOSTICS

Neela Prajapati[1†], Anik Karan[1†], Elnaz Khezerlou[1], and Mark A. DeCoster[1,2*]

[1]Department of Biomedical Engineering, Louisiana Tech University, Ruston, LA 71270, USA.

[2]Institute for Micromanufacturing, Louisiana Tech University, Ruston, LA 71270, USA.

***Correspondence:**
Mark A. DeCoster
decoster@latech.edu

† These authors have contributed equally to this work.

Treatment of CuHARS (10 µg/ml) in presence of CysNO in BMVECs

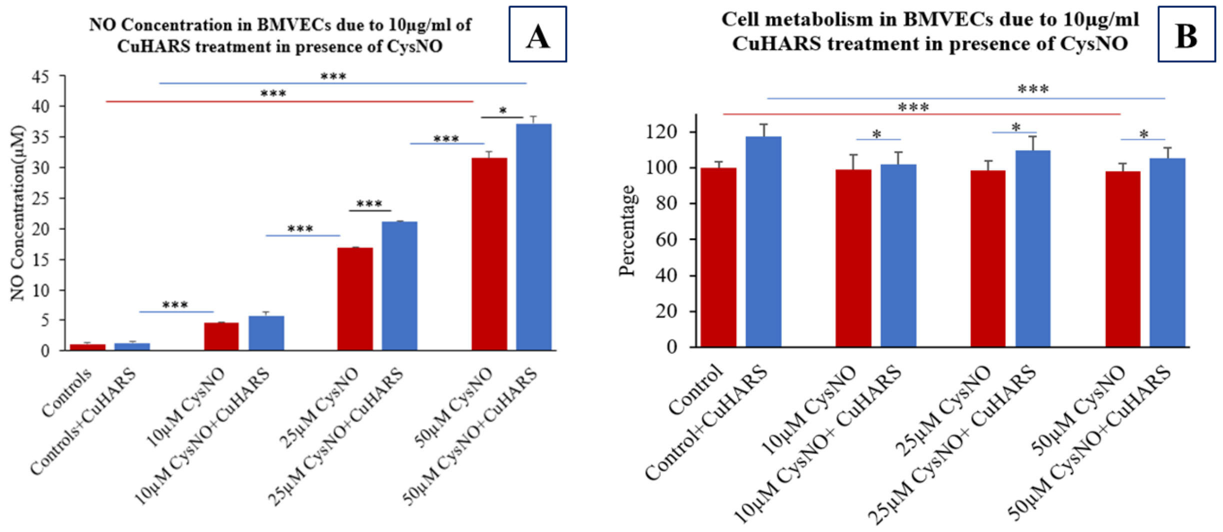

Supplementary Figure 1 | NO response of normal brain endothelial cell (BMVECs) to low concentration of CuHARS (10µg/ml) in presence of three different concentrations (10,25 and 50 µM) of CysNO, an NO precursor normally found in blood. A) NO concentration in the BMVECs due to the treatment showing significant increase in NO release from cells treated with CuHARS compared to cells treated with CysNO alone B) Cell metabolism due to the treatment showing increase in cellular metabolism for CuHARS treated cells compared to when treated with CysNO alone. Data represent average of three experiments(N=3) with triplicated wells (n=3). The error bars represent SEM values, "***" represents $p<0.001$ and "*" represents $p<0.05$.

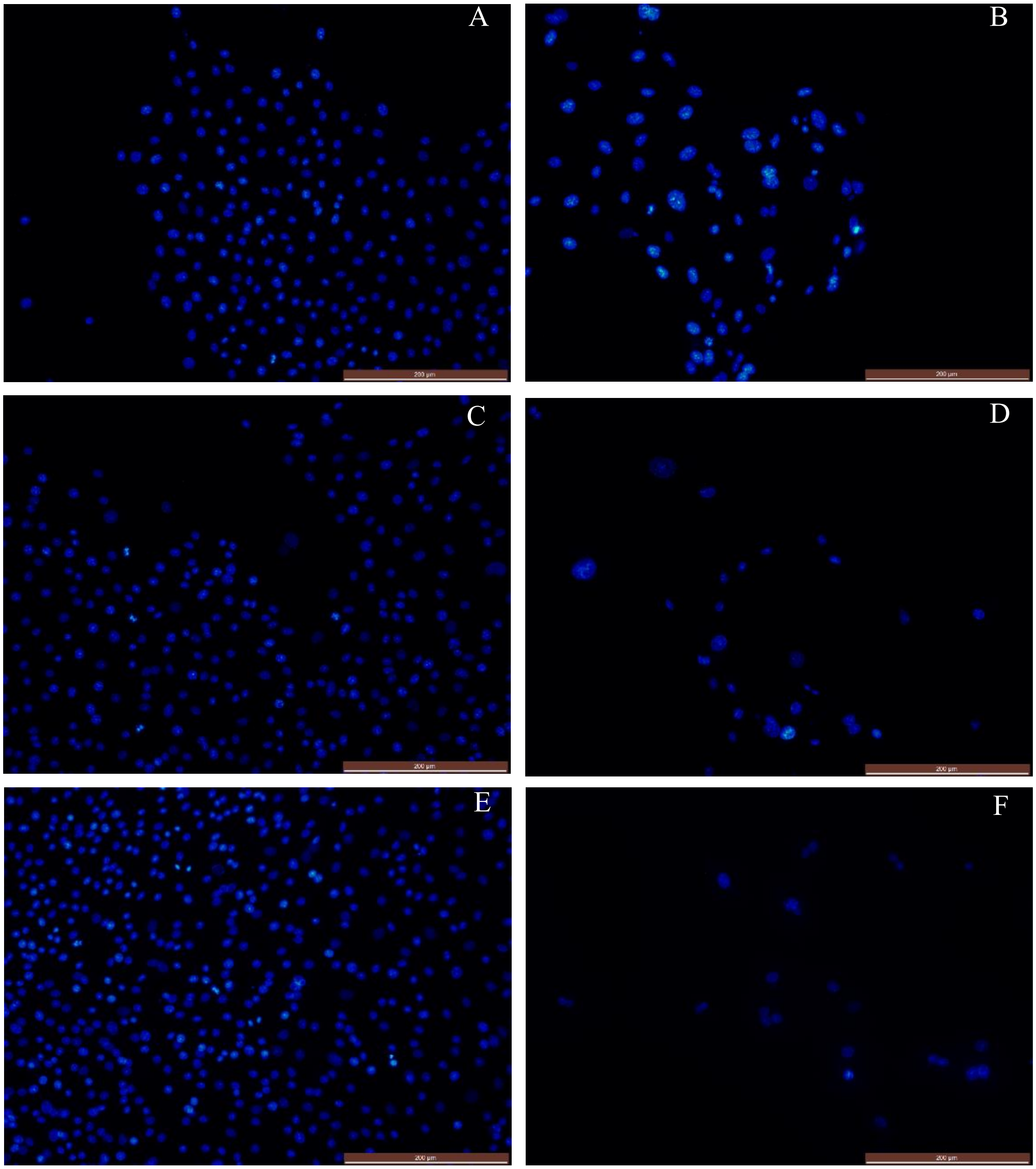

Supplementary Figure 2 | DAPI stained images of glioma (CRL2303) cells plated at 3,000 per ml density for **Controls** (Right) and **Treated** (Left) showing increase in toxicity with successive treatments of 1 dose [A-B], 2 doses [C-D] and 3 doses [E-F] of 10 µg/ml of CuHARS. Magnification 200X, Scale bar =200µm.

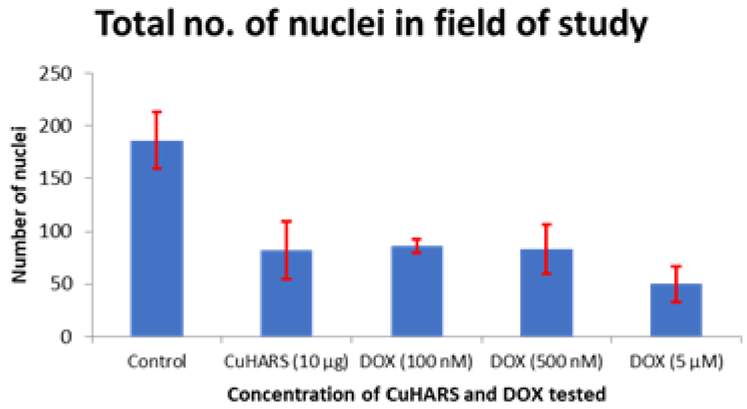

Supplementary Figure 3 | DAPI analysis for total count of glioma (CRL2303) cells plated at 3,000 per mL density treated with 10 µg/ml of CuHARS and different concentrations of Doxorubicin.
